# Differential impacts of vaccine scandal by ethnic and socioeconomic factors: Evidence from China

**DOI:** 10.1371/journal.pone.0288841

**Published:** 2023-07-19

**Authors:** Mengna Luan, Qi Qi, Wenjing Shi, Zhigang Tao, Ying Bao, Jiushun Zhou

**Affiliations:** 1 Research Institute of Economics and Management, Southwestern University of Finance and Economics, Chengdu, China; 2 Sichuan Center for Disease Control and Prevention, Chengdu, China; 3 Faculty of Business and Economics, The University of Hong Kong, Hong Kong Special Administrative Region, China; 4 Cheung Kong Graduate School of Business, Beijing, China; Sichuan Agricultural University, CHINA

## Abstract

Widespread vaccination against important diseases plays a key role for global health security, particularly in the context of the COVID-19 pandemic. However, building and maintaining trust in immunization services remains challenging because of doubts about quality and safety of vaccines. China has periodically faced mounting pressure and even public outrage triggered by incidents of poor-quality vaccines. We aimed to evaluate the impact of the diphtheria, pertussis, and tetanus (DPT) vaccine scandal of 2018 in China and the ensuing misinformation on vaccination, and investigate differential responses to the scandal by ethnic and socioeconomic factors. With data from January 2017 to December 2018 in Sichuan province, China, we used a difference-in-differences (DID) method to compare the changes in the county-level monthly DPT vaccinations against the hepatitis B vaccinations, both before and after the DPT vaccine scandal. We found that the number of DPT vaccinations decreased by 14.0 percent in response to the vaccine scandal and ensuing misinformation. The number of vaccinations in minority regions, under-developed regions, and regions with poor medical resources decreased more than in non-minority regions, developed regions, and regions with good medical resources (24.5 versus 10.1 percent, 17.3 versus 8.3 percent, and 17.0 versus 8.7 percent, respectively). People did more online searching for “Substandard vaccine” and “DPT vaccine” after the scandal, with the socioeconomically advantaged group searching more compared with the socioeconomically disadvantaged group. The results suggest the urgent need to make true information about the vaccine easily accessible over the internet, especially for the socioeconomically disadvantaged groups. Our findings for China can also have implications for immunization service planning for better safeguarding public health in other countries, particularly developing ones.

## Introduction

Vaccination saves millions of lives around the world every year. Yet, universal uptake of basic vaccines has been a challenging task [1]. In 2020, an estimated 23 million children under the age of 1 year did not receive basic vaccines [2]. Causes of vaccine hesitancy are complex, ranging from spread of misinformation (often triggered by quality and efficacy issues relating to vaccines) on one hand, and lack of effectiveness public health messaging on the other hand, and they may have differential impacts on people of different ethnic and socioeconomic factors. Despite anecdotal evidence suggesting the importance of misinformation and its varying impacts across different groups of people there is a lack of systemic studies on these causes of vaccine hesitancy, especially in developing countries where such causes are of more prominent importance. This study is to fill in this research gap by examining the impact of misinformation on vaccine hesitancy in a developing country, and exploring if such impact varies across group of people with different ethnic and socioeconomic backgrounds.

This study utilized one of the largest and most well-known vaccine scandals in China. In July 2018, news broke out that two vaccine producers, Changchun Changsheng Biotechnology (a listed company) and Wuhan Institute of Biological Products, had produced and distributed 499,800 and 400,520 substandard diphtheria, pertussis, and tetanus (DPT) vaccines, respectively, in four regions in China [3]. Although the vaccines were ineffective rather than harmful with no report of death or other severe consequences, misinformation about the substandard vaccines being toxic went viral on social media way beyond the four regions that were supplied and actually affected by the two producers. The scandal and the ensuing misinformation sparked widespread anger and panic, causing public distrust in vaccination [4–7]. More details on the scandal and the timeline are shown in S1 Table and S1 Fig.

To investigate the impact of misinformation of vaccine safety and effectiveness, we examined vaccination of DPT shots in Sichuan (a Chinese province that was not supplied by the two problem vaccine makers) versus the vaccination of hepatitis B (which serves as a control), both before and after the aforementioned vaccine scandal relating to DPT. Moreover, utilizing the variations across 183 countries/districts within Sichuan province, we examined differential responses to the scandal by ethnic and socioeconomic factors such as ethnic group composition, GDP per capita, and medical resources, and we tried to link the differential responses to the abilities by people in different regions in discerning the truthful information about the vaccine scandal (proxied by the use of internet search intensity over Baidu—a dominant Chinese search engine—of the scandal, or the daily Baidu search index of this scandal).

This study departs from previous studies in three ways, while there is a growing literature on the negative effects of vaccine scandals/incidents on vaccination coverage in developed and developing countries [8–14]. First, to our knowledge, this is the first study on the differential impacts of the misinformation relating to a vaccine scandal on vaccinations by ethnic and socioeconomic factors in China. Second, adopting a natural experimental design of the vaccine scandal event while controlling for general time varying changes through the use of the DID approach, this study is based on a more stringent causal inference analysis. Last, we examined the internet search behaviors to understand the potential reasons behind the differential effects of the vaccine scandal across different groups of people, which highlights the importance of information disclosure as a way of battling against misinformation about vaccine safety and efficacy and improving immunization uptake in the digital era.

## Methods

### Background of Sichuan province

In this study, we used data from Sichuan, a province with a population of more than 80 million people in southwestern China, for two reasons. First, Sichuan was not one of the destination markets of the substandard DPT vaccines; therefore, the province was not affected by re-vaccination in the post-scandal period for children who had received substandard DPT vaccines. Anhui province, Chongqing city (under the direct administration of the central government), Hebei province, and Shandong province are the four and only four regions that received the substandard DPT vaccines. The second reason is that, Sichuan has large within-province variations. The Heihe-Tengchong line divides China and Sichuan into two roughly equal areas. The area to the east of the line has much lower elevation than the area to the west of the line. Sichuan also has large variations in population density, level of economic development, ethnic composition, and medical resources. Specifically, population is concentrated in the eastern part of the province; GDP per capita is also generally higher in the eastern part; ethnic minorities live in the western part; medical resources, as proxied by number of hospital beds per 1,000 persons, are concentrated in the eastern part. The large variations allow us to explore the possibly differential impacts of the misinformation about the vaccine scandal. Moreover, Sichuan’s relative ranking among provinces in population and gross domestic product (GDP) per capita were detailed in S2 Fig.

### Data source and variables

Our main data set covered the monthly number of vaccinations for DPT and hepatitis B across all counties/districts in the entire Sichuan province from January 2017 to December 2018. There are 183 counties/districts governed by 21 cities in the province. The data were collected and audited monthly by the Sichuan Center for Disease Control and Prevention (CDC). In China, rural areas are organized at the county level, while urban areas are organized at the district level. Counties and districts are at the same administrative level, and they are under the administration of cities. We used the term county-level in this paper for simplicity.

DPT vaccines were involved in the vaccine scandal, but hepatitis B vaccines were not. Both vaccines are classified as the Expanded Program on Immunization (EPI) vaccines by the Chinese central government [15]. All vaccines in EPI are provided to citizens free of charge. For DPT vaccination, infants get three shots in the first year (at ages three, four, and five months) and a booster shot at eighteen months. Infants are also vaccinated for hepatitis B within 24 hours of birth, as well as at one and six months. For newborns, vaccinations are normally performed right after delivery in the hospital, and hospitals report the numbers of vaccination to local (county-level) CDCs on a regular basis in China. As the first shot of hepatitis B is mandatory, there is little variation in the number of vaccinations. Therefore, in this study, we focused on the second and third hepatitis B shots and all the DPT shots.

We used the county-level monthly number of vaccinations as our first outcome variable. The average monthly number of vaccinations is 1,524 for DPT vaccine and 804 for hepatitis B vaccine on average. We could not use the vaccination rate, because monthly data on the number of ready-for-vaccination infants (the eligible population) were not observed in the data. However, given that the monthly number of ready-for-vaccination infants remained stable in the short run after controlling for seasonal factors, changes in the monthly numbers of vaccinations would capture the responses of the public to the vaccine scandal and the ensuing misinformation. We also collected the county-level annual number of newborns to account for the potential time trends of the number of ready-for-vaccination infants in the medium and long run.

Moreover, we used a set of county-level data from 21 city statistical yearbooks, bulletins in Sichuan, and Sichuan CDC as control variables. First, to control for factors that might have affected the number of vaccinations, we included (logarithm) population and (logarithm) GDP per capita (monthly data for these variables were not reported publicly). Second, we also included the number of hospital beds per 1,000 persons to account for variations in medical resources across counties. Lastly, we included the monthly supply of the vaccines administered by Sichuan CDC, to control for variations in vaccine stocks, as lower vaccine supply might have led to the provision of fewer vaccinations. Note that there were missing county-level numbers of newborns for seven counties in 2017 and 2018. We used city-level number of newborns to multiply the county’s share in the city’s population instead. In addition, the county-level number of beds data were missing for five counties in 2018, so we used city-level number of beds instead to calculate number of beds per 1,000 persons.

We used the Baidu search index for vaccines to capture the public’s information search behaviors via internet in response to the vaccine scandal as another outcome variable in this study. The Baidu search index is an online search index that measures the popularity of search queries. We chose this search index because Baidu is the largest web search engine in China and Baidu search index has been widely used to capture the internet search behavior [5, 16, 17]. Using Baidu, we searched for the terms “Substandard vaccine” (“Wenti Yimiao” in Chinese) and “DPT vaccine” (“Baibaipo Yimiao” in Chinese). We obtained the index of searching trends on personal computers and mobile phones from July 14, 2018 to July 28, 2018 on a daily basis for each city in Sichuan province. In this sample period, the index is 0.99 for “DPT vaccine” and 1.10 for “Substandard vaccine”. In the analysis of differential effects on the Baidu search index, we used three variables to capture the socioeconomic features of the cities, namely GDP per capita, the number of beds per 1,000 persons, and a dummy variable for non-minority status. The non-minority status dummy equaled 1 if the region is predominantly home to Han ethnic groups, and 0 otherwise. The city-level population and GDP data were aggregated from the county-level data. The data on city-level number of hospital beds per 1,000 persons were obtained from the 2018 Sichuan Provincial Statistical Yearbook.

Descriptive statistics for the data and variables are presented in Table 1.

**Table 1:** Descriptive statistics.

**Panel A. No. vaccinations (doses)**	Observations	Mean	S.D.
DPT (Treatment group)	4,464	1524.24	1466.77
Hepatitis B (Control group)	4,464	803.56	731.24
**Panel B. Baidu search index**	Observations	Mean	S.D.
DPT vaccine	315	0.99	2.18
Substandard vaccine	315	1.10	2.88
**Panel C.**	Observations	Mean	S.D.
*For county-level analysis*			
Population	8,784	450754.9	320112.1
GDP per capita (in RMB)	8,784	41979.55	26222.87
No. newborns	8,784	5146.81	4104.08
Minority status	8,928	0.27	0.45
No. hospital beds per 1,000 persons	8,784	6.38	2.91
Vaccine supply (doses)	8,778	1166.05	1656.94
*For city-level analysis*			
GDP per capita (in RMB, 0’000)	315	4.09	1.79
Non-minority status	315	0.86	0.35
No. hospital beds per 1,000 persons	315	6.52	1.10

### Statistical analysis

#### Difference-in-Differences (DID) estimation

**Fig 1:**
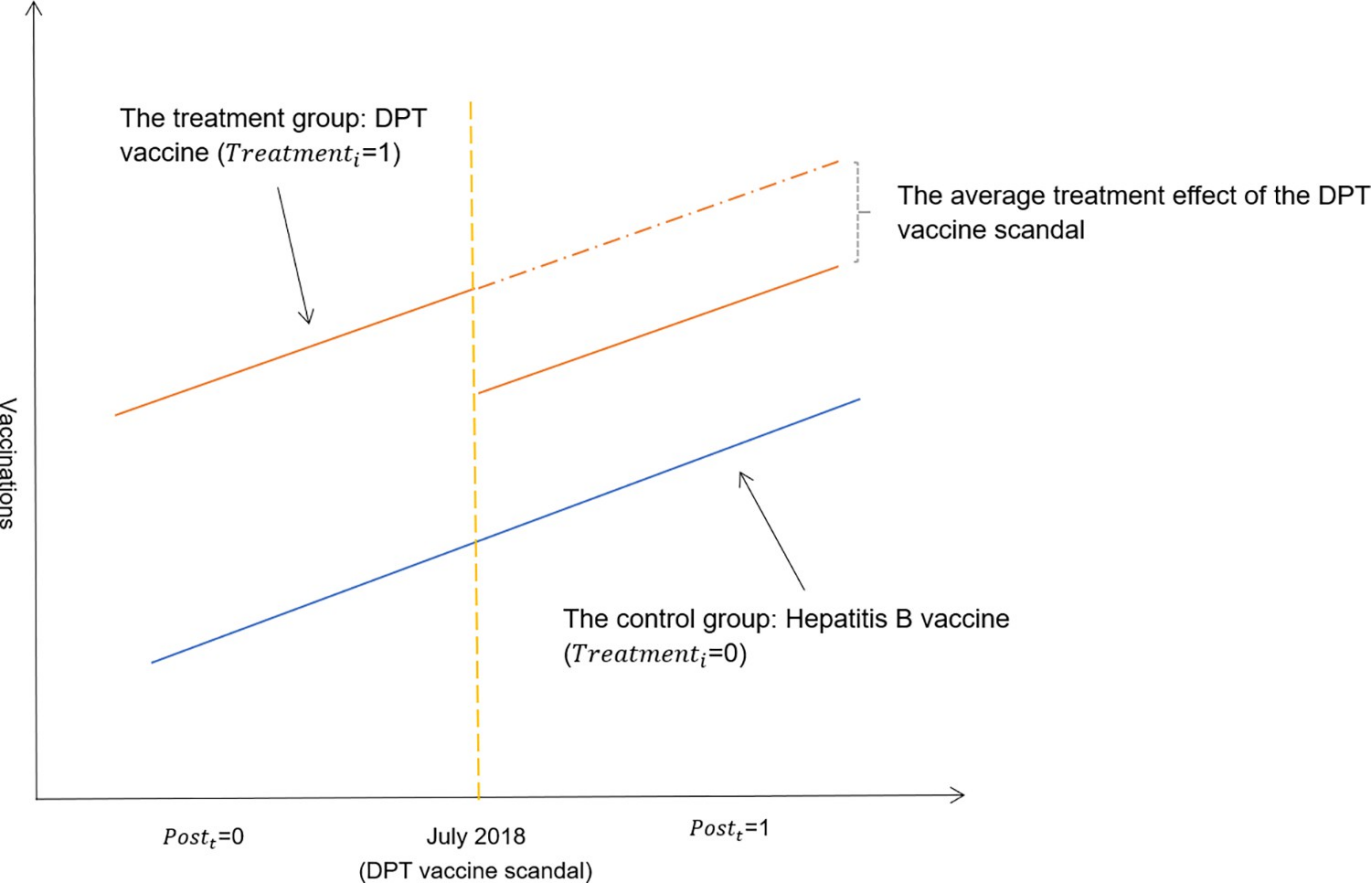
The graphical illustration of the DID model setting used in this study.

We estimated the impact of the vaccine scandal and ensuing misinformation on the number of vaccinations using the county-level monthly data. The most intuitive way to investigate the impact of a vaccine scandal is to conduct before-after comparisons. However, vaccinations often have seasonal patterns or other time trends driven by unobserved confounding factors, and therefore the causal impact of a vaccine scandal on vaccinations cannot be inferred simply by performing before-after comparisons. To address issues like the confounding factors, we employ the DID approach, which is to take care of the general time trend and has been widely used in the literature [18–22]. In our DID estimation model, we compared the change in the outcome over the sample period between the treatment group of DPT vaccine, which were the target during the DPT vaccine scandal, and the control group of hepatitis B vaccine, which were not affected by the scandal, both before and after the vaccine scandal event. Fig 1 presents a graphical illustration of the DID model setting used in this study. Specifically, the DID model for the number of vaccinations is as follows:

$$y_{ikt}=\beta {Treatment}_{i}*{Post}_{t}+X_{kg}\eta +\omega_{ikt}+\theta_{i}+\delta_{k}+\mu_{t}+\epsilon_{ikt}$$ (1)

where *y*_*ikt*_ denotes (logarithm) number of vaccinations of vaccine type *i* (namely, DPT or hepatitis B) in county *k* in month *t*. *Treatment*_*i*_ is a dummy variable that equals 1 if the vaccine type is DPT, and 0 if the vaccine type is hepatitis B. *Post*_*t*_ is a dummy variable that equals 1 for vaccinations after July 2018, and 0 otherwise. **X**_*kg*_ is a vector of observed characteristics in county *k*, in year *g*, including (logarithm) population, (logarithm) number of newborns, (logarithm) GDP per capita, and (logarithm) number of beds per 1,000 persons. We also considered the monthly supply of the vaccine to control for variations in vaccine stocks. As the vaccine supply should have a delayed effect on the number of vaccinations, we control for the lagged vaccine supply of vaccine type *i* in county *k* by summing up the previous three-month supply ($\omega_{ikt}=\sum_{j=t-3}^{t-1}\sigma_{ikj}$, where *σ*_*ikj*_ denotes the supply of vaccine type *i* in county *k* in month *j*). *θ*_*i*_ is the vaccine fixed effects capturing the characteristics of vaccines that do not change over time. *δ*_*k*_ is the county fixed effects capturing all the time-invariant characteristics of the counties, which might influence the outcome of interest. *μ*_*t*_ represents the year-month fixed effects, controlling for province-wide shocks in a particular month of a particular year that may have affected all counties in a similar manner. *ϵ*_*ikt*_ is the error term representing any unobserved shocks to the dependent variable. We accounted for the clustered nature of the data by constructing standard errors corrected for county level clustering. *β* is the coefficient of interest, which represents the change of the number of vaccinations due to the scandal and ensuing misinformation.

We further examined the differential effects of the scandal and ensuing misinformation on the number of vaccinations, by conducting three subsample analyses using the same DID model (Eq 1). The three subsample grouping factors are ethnic groups, GDP per capita, and medical resources. Specifically, minority regions are counties that are predominantly home to non-Han ethnic groups. We categorized the counties as under-developed regions, if their 2017 GDP per capita was less than the mean value in Sichuan province, which was 40,518 RMB per capita. If the 2017 county-level number of hospital beds per 1,000 persons was less than the mean value in Sichuan province, which was 6.11 beds per 1,000 persons, we considered these counties as regions with poor medical resources. We then conducted the DID estimation for each subsample using Eq (1) and compared the results within each pair (minority vs. non-minority regions, under-developed vs. developed regions, and regions with poor medical resources vs. regions with good medical resources).

#### Event study

To understand the potential reasons behind the differential effects of the vaccine scandal and the ensuing misinformation across different groups of people, we used the event study method to assess how the vaccine scandal affected the daily Baidu search index of “Substandard vaccine” and “DPT vaccine” at the city-level. The method is widely used to identify sudden and statistically significant reactions to past occurrences of the event of interest and is particularly suitable for short-horizon analysis [23, 24]. In our context, the event study approach can elicit the influences of the vaccine scandal observed over a short time window. On July 20, 2018, China’s Jilin Provincial Medical Products Administration accused Changchun Changsheng Biotechnology of selling substandard DPT vaccines. As the media began to report extensively on the punishment announcement on July 21, 2018, we considered that the event of the vaccine scandal took place on July 21, 2018. We focused on the data period from July 14, 2018 to July 28, 2018, a 14-day time window.

Using the event study method, we first analyzed the public’s internet information search behaviors in response to the vaccine scandal and ensuing misinformation, using Eq (2) listed below:

$$y_{ct}=\alpha_{-7}D_{t}^{-7}+\alpha_{-6}D_{t}^{-6}+\alpha_{-5}D_{t}^{-5}+\alpha_{-4}D_{t}^{-4}+\alpha_{-3}D_{t}^{-3}+\alpha_{-2}D_{t}^{-2}+\alpha_{-1}D_{t}^{-1}+\alpha_{1}D_{t}^{1}+\alpha_{2}D_{t}^{2}+\alpha_{3}D_{t}^{3}+\alpha_{4}D_{t}^{4}+\alpha_{5}D_{t}^{5}+\alpha_{6}D_{t}^{6}+\alpha_{7}D_{t}^{7}+\delta_{c}+\epsilon_{ct}$$ (2)

where *y*_*ct*_ denotes the Baidu search index of “Substandard vaccine” or “DPT vaccine” in city *c* on day *t*. To check the time trends, we included $D_{t}^{j}$ which indicates the relative sequence of day *t* subject to the scandal time of July 21, 2018. Specifically, when *j* = -1 to -7, $D_{t}^{j}$ equals 1 for 1^st^ to 7^th^ days before the scandal time, and 0 otherwise; when *j* = 1 to 7, $D_{t}^{j}$ equals 1 for 1^st^ to 7^th^ days after the scandal time, and 0 otherwise. $D_{t}^{0}$ (the day of scandal) is omitted as the benchmark day. We included the city fixed effects (*δ*_*c*_) in the model. *ϵ*_*ct*_ is the error term representing any unobserved shocks to the dependent variable, the Baidu search index. If *α*_*j*_ (*j* = -1 to -7), the coefficients of interest for the days before the scandal are insignificant, then there are no differences in the search index for the two search terms between the pre-scandal period and the benchmark day, which would support the validity of our event study analyses.

We then examined how ethnic, economic, and medical resource factors affected internet searching on the vaccine scandal within the event study setting. The regression models are listed below numbered (3), (4), and (5):

$$y_{ct}=\gamma_{1}{NMinority}_{c}*{Post}_{t}+\zeta_{1}{NMinority}_{c}+\lambda_{1}{Post}_{t}+\delta_{c}+\epsilon_{ct}$$ (3)

$$y_{ct}=\gamma_{2}{GDP}_{c}*{Post}_{t}+\zeta_{2}{GDP}_{c}+\lambda_{2}{Post}_{t}+\delta_{c}+\epsilon_{ct}$$ (4)

$$y_{ct}=\gamma_{3}{Beds}_{c}*{Post}_{t}+\zeta_{3}{Beds}_{c}+\lambda_{3}{Post}_{t}+\delta_{c}+\epsilon_{ct}$$ (5)

where *y*_*ct*_ denotes the Baidu search index of “Substandard vaccine” or “DPT vaccine” in city *c* on day *t*. *NMinority*_*c*_ represents the dummy variable that equals 1 if the region is predominantly home to national majority Han ethnic group, and 0 otherwise in Eq (3). *GDP*_*c*_ indicates the GDP per capita of year 2017 for city *c* and *Beds*_*c*_ indicates the number of hospital beds per 1,000 persons of 2017 for city *c* in Eq (4) and Eq (5), respectively. *Post*_*t*_ is a dummy variable that equals 1 if the day is after July 20, 2018, and 0 otherwise. We also included the city fixed effects (*δ*_*c*_). *ϵ*_*ct*_ is the error term representing any unobserved shocks to the dependent variable, the Baidu search index. *γ*_1_, *γ*_2_, and *γ*_3_ are the coefficients of interest, which capture the heterogeneous effects of the scandal on the Baidu index.

This study was approved by University of Hong Kong Human Research Ethics Committee (EA200205).

## Results

In this section, we first reported the results of the DID estimation in Table 2. Our outcome variable is the log transformation of number of vaccinations, and the coefficient of *Treatment*_i_*Post_*t*_ can be interpreted as percentage changes. As shown in the first column in Table 2, the number of vaccinations for DPT decreased by 14.0 percent in response to the vaccine scandal and ensuing misinformation. This indicates an average reduction in the monthly number of vaccinations by 223 in a county, given the monthly mean number of DPT vaccinations prior to the scandal at the county level being 1,590. We found similar results with an alternative specification which excluded the vaccine supply as control (see S2 Table). Details concerning the robustness check on parallel trend assumption for DID estimation are presented in S3 Fig.

**Table 2:** Changes in number of vaccinations in response to the vaccine scandal and ethical and socioeconomic subsample analysis.

	(1)	(2)	(3)	(4)	(5)	(6)	(7)
Number of vaccinations (logarithm)	Whole sample	Minority regions		Under-developed regions		Regions with poor medical resources	
		YES	NO	YES	NO	YES	NO
Treatment* Post	-0.140 (-0.178 to -0.103)	-0.245 (-0.344 to -0.146)	-0.101 (-0.136 to -0.066)	-0.173 (-0.227 to -0.119)	-0.083 (-0.128 to -0.039)	-0.170 (-0.222 to -0.118)	-0.087 (-0.137 to -0.037)
P value	0.000	0.000	0.000	0.000	0.000	0.000	0.001
Observations	7,460	2,040	5,420	4,651	2,809	4,749	2,711
R-squared	0.940	0.870	0.953	0.917	0.972	0.929	0.949
Z-score	NA	-2.76 (P value 0.006)		-2.56 (P value 0.010)		-2.30 (P value 0.022)	

*Notes*: All regressions include county, vaccine type, and year-month fixed effects, as well as control variables for population, number of newborns, vaccine supply, GDP per capita, and number of hospital beds per 1,000 persons. Heteroskedasticity robust standard errors are clustered at the county level. 95% confidence intervals of *Treatment*_i_*Post_*t*_ are reported in parentheses.

Next, we investigated the heterogeneous effects of the scandal on the number of vaccinations, by conducting three subsample analyses: minority versus non-minority regions, under-developed versus developed regions, and regions with poor medical resources versus regions with good medical resources. As shown in Table 2 columns (2) and (3), the number of vaccinations for DPT decreased by 24.5 percent in minority regions but by 10.1 percent in non-minority regions. The number of vaccinations for DPT decreased by 17.3 percent in under-developed counties (column (4)) but by 8.3 percent in developed counties (column (5)). Last, the number of vaccinations for DPT decreased by 17.0 percent in regions with poor medical resources (column (6)) but by 8.7 percent in regions with good medical resources (column (7)). The z-score values showed that the changes in the numbers of vaccinations due to the scandal for each pair of subsamples are all statistically different. The results indicated that people living in socioeconomically disadvantaged areas reacted more strongly to the vaccine scandal and the ensuing misinformation by refusing or delaying vaccinations for their children. We found similar results with an alternative categorization of regions (S3 and S4 Tables).

**Fig 2:**
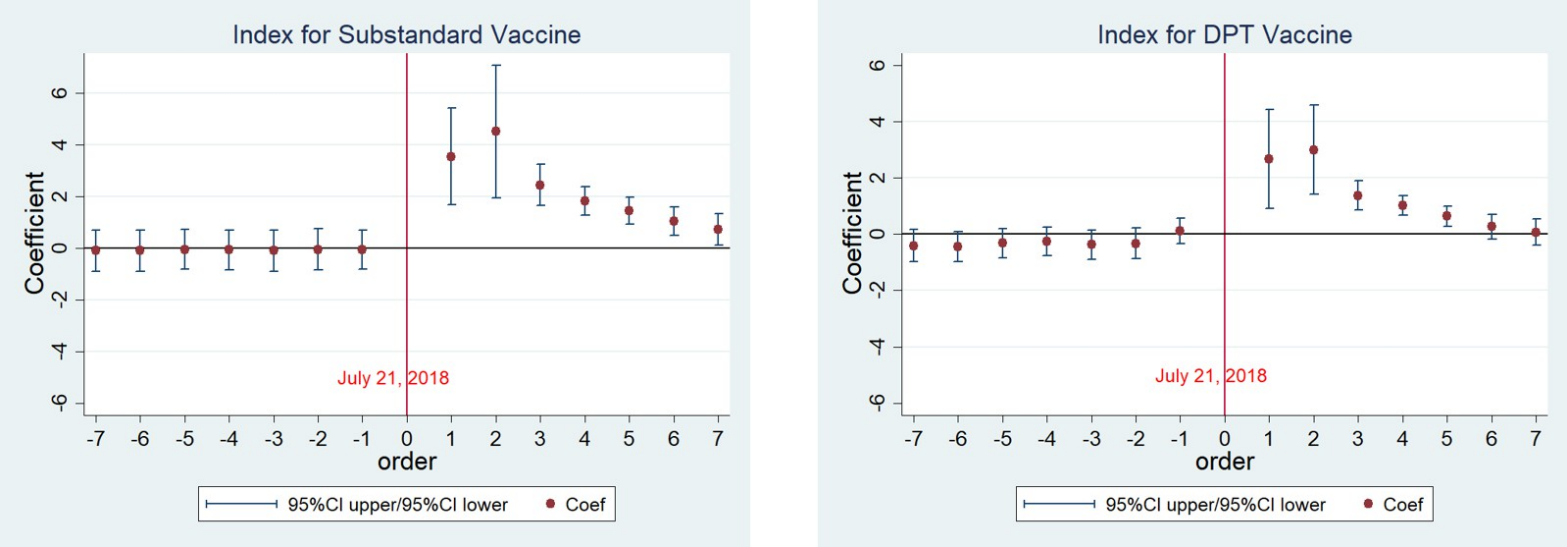
Event study analysis for online searches of “Substandard vaccine” and “DPT vaccine”.

To understand the differential effects of the scandal and the misinformation across different groups of people, we examined the public’s internet information search behaviors in response to the scandal, by combining the data set of the Baidu search index. The search index reflects the popularity of search queries, and has no units. Prior to the scandal, the mean of search indexes for “Substandard vaccine” and “DPT vaccine” were 0.02 and 0.23, respectively. As shown in Fig 2, for “Substandard vaccine” and “DPT vaccine,” the coefficients of the daily dummy variables prior to the scandal were all insignificant, indicating no differences in the search index between the pre-scandal period and the benchmark day (July 21, 2018) for the two search terms; however, the coefficients of the days after the scandal become significantly positive, showing dramatic increases in the search index for the two search terms over the post-scandal period compared with the benchmark day. The event study analysis indicated that the public searched online significantly more for the search terms “Substandard vaccine” and “DPT vaccine” after the scandal.

Within the event study setting, we further examined how socioeconomic and medical resource factors affected the internet search behaviors in the post-scandal period. We reported the results in Table 3. After the scandal, the Baidu search index for “Substandard vaccine” and “DPT vaccine” in non-minority regions increased 1.14 (panel A, column (1)) and 1.05 (panel B, column (1)) more than that in minority regions, respectively. In addition, along with 10,000 RMB (equivalent to $1,484 USD using 1:6.74 exchange rate as of July 31, 2022) increase in GDP per capita, the Baidu search indexes for “Substandard vaccine” and “DPT vaccine” increased more by 0.81 (panel A, column (2)) and 0.53 (panel B, column (2)), respectively. Last, along with 1 hospital bed per 1,000 persons increase, the Baidu search indexes for “Substandard vaccine” and “DPT vaccine” increased more by 0.97 (panel A, column (3)) and 0.67 (panel B, column (3)), respectively. Given that the mean of Baidu search indexes for “Substandard vaccine” and “DPT vaccine” before the scandal were 0.02 and 0.23, respectively, the results showed that there was a large increase in internet search increase after the DPT vaccine scandal but the increase was substantially less among those living in socioeconomically disadvantaged areas, indicating that they may have received less detailed and accurate information on the DPT vaccine scandal.

**Table 3:** Heterogeneous impacts of the scandal on the Baidu search index.

	(1)	(2)	(3)
**Panel A. Keyword: Substandard vaccine**			
NMinority*Post	1.138 (0.467 to 1.810)		
GDP*Post		0.806 (0.155 to 1.456)	
Beds*Post			0.970 (0.247 to 1.693)
P value	0.001	0.015	0.009
Observations	315	315	315
R-squared	0.364	0.422	0.394
**Panel B. Keyword: DTP vaccine**			
NMinority*Post	1.051 (0.554 to 1.548)		
GDP*Post		0.533 (0.049 to 1.017)	
Beds*Post			0.669 (0.135 to 1.203)
P value	0.000	0.031	0.014
Observations	315	315	315
R-squared	0.393	0.434	0.414

*Notes*: All the results include city fixed effects. 95% confidence intervals are reported in parentheses, which are based on robust standard errors.

## Discussion

Our study provided new evidence on the impact of China’s vaccine scandal on vaccination, by analyzing a unique, county-level monthly data set for a southwestern province in the country. The study found the 2018 vaccine scandal and ensuing misinformation were associated with the decreased numbers of DPT vaccinations (14.0 percent decline) and the decline was larger in minority regions, under-developed regions, and regions with poor medical resources, relative to non-minority regions, developed regions, and regions with good medical resources (24.5 versus 10.1 percent, 17.3 versus 8.3 percent, and 17.0 versus 8.7 percent, respectively).

To our knowledge, this study is the first evaluation of the differential impacts of a vaccine scandal and the ensuing misinformation on vaccinations by ethnic and socioeconomic factors in China. As vaccine hesitancy is associated with the epidemiology of vaccine-preventable diseases [25], our results indicated that the minority regions, under-developed regions, and regions with poor medical resources were exposed to higher risk of disease outbreaks. What is worse, the lower level of economic development and less access to health care services in those regions would make people there more vulnerable to the risk.

One possible explanation for the differential responses across groups of parents to the scandal is that people in regions with higher level of economic development and better access to health care services might have been more inclined to do their own research to figure out the whole picture of the vaccine scandal despite the fake stories of “toxic vaccines” going viral on social media. Thus, those people may have been less fearful and less likely to hold back their children from being vaccinated. That is, they knew that, despite the scandal, substandard DPT vaccines were ineffective rather than harmful and more importantly the batches of substandard DPT vaccines did not affect Sichuan province at all. Our findings about the Baidu index supported this explanation: people in non-minority regions, more developed regions, and regions with better medical resources were more likely to actively search information about the DPT vaccine scandal online using Baidu (Table 3). The differential effects of the scandal on vaccination and internet information searches together indicated that online information plays an important role when people make decisions on vaccination for their children in the digital era. Hence, it is critical to refute misinformation and make true information easily accessible over the internet.

In addition to the literature on the impact of vaccine scandals/incidents on vaccination, this study contributes to the literature on differential behavioral responses to misinformation on vaccine safety among different groups of people. Most existing studies focus on the measles‐mumps‐rubella (MMR)—autism controversy in developed countries, and find differential responses among groups of parents by education. For the U.K., previous analyses show that the uptake rate of the MMR in the wake of the controversy declined faster in areas where a larger fraction of parents had stayed in education past the age of 18 than in areas with less educated parents [26]. For the U.S., recent studies provide evidence that more highly educated mothers responded more strongly to the controversy either by not immunizing their children or delaying vaccination [27, 28].

This study complements these existing studies in two important ways. First, our study finds differential responses among groups of parents by ethnic group status, income, and access to health care services, while the previous studies focus on differential behaviors among groups of parents by education. Second, this study finds that the socioeconomically disadvantaged group of parents (i.e., those living in minority regions, under-developed regions, and regions with less access to health care services) in a large developing country responded more negatively to a vaccine scandal and the ensuing misleading information by delaying/refusing vaccination services for their children, while the previous studies find that more educated parents in developed countries had stronger responses. The different patterns in developed and developing countries poses significant challenge of developing strategies to battle vaccine hesitancy globally.

In the context of the COVID-19 pandemic, international organizations and governments played an active role in debunking rumors and updating real information on the internet during the crisis. For example, World Health Organization homepage lists detailed COVID-19 information, including vaccines, scam alert, etc. U.S. Food and Drug Administration homepage shows the rigorous scientific and regulatory documents for COVID-19 vaccines to ease public concerns. U.K. National Health Service homepage provides guidance for the public to understand COVID-19 test results and search for COVID-19 tracing system. All these websites appear at the top of Google search outcomes when searching for “COVID vaccine” or “COVID vaccine side effects”. Nevertheless, lessons from the Chinese case discussed in this study urge us to do more, because certain groups of people may not actively search for real and comprehensive information with search engines. These groups of people are thus more likely to be influenced by fake news or stories circulated on social media. An effective rumor-refuting approach in the digital era should emphasize the role of social media in providing access to real information to more disadvantaged people.

Our study has two limitations. One concern is that the number of vaccinations for hepatitis B might also have been affected by the DPT vaccine scandal. If this was the case, our results would still be able to provide a lower bound estimate for the public’s reactions to the scandal. Next, the vaccine scandal took place in July 2018 and thus the post-scandal data period in our study which covers only August to December 2018, might not be long enough to fully capture the public’s behaviors after the scandal. We can observe the short-term effects of vaccination reduction, but we are not able to examine whether there would be a rebound so that the DPT vaccinations can get recovered in the long run. The knowledge gap on the long-run impact of the scandal cannot be overemphasized and must be addressed in the future when more data are available.

## Conclusion

Our findings show that the number of vaccinations will go down in response to a trust-undermining vaccine scandal, in particular among those living in socioeconomically disadvantaged areas. To ensure universal childhood vaccination, China’s local CDCs and community clinics, which are in charge of vaccinations, should pay more attention to the socioeconomically disadvantaged groups (ethnic minority groups and lower income groups), and address their considerations to maintain their trust in vaccination in the event of a vaccine scandal. With the advancement of digital technologies (e.g., mobile phones and social media), health authorities should be transparent in releasing health information, for example, on vaccine scandals. In addition to traditional forms, e-forms of promoting and marketing vaccinations should be in place. More importantly, in the digital era, the role of social media in refuting rumors and providing access to real information cannot be overemphasized.

## Supporting information

S1 Fig Scandal timeline. (PDF)

S2 Fig Population and GDP per capita comparison between Sichuan and other provinces in China, 2018. (PDF)

S3 Fig Parallel trends assumption check for number of vaccinations. (PDF)

S1 Table The vaccine scandal in numbers, 2018. (PDF)

S2 Table Baseline estimation and estimation without vaccine supply as control. (PDF)

S3 Table List of impoverished counties in Sichuan. (PDF)

S4 Table Estimation for impoverished and non-impoverished counties. (PDF)

S1 Data (DTA)

S2 Data (DTA)

S3 Data (DTA)

S4 Data (DTA)
