# Supplementary material for: Differential impacts of vaccine scandal by ethnic and socioeconomic factors: Evidence from China
Source: PLoS One. 2023 Jul 19;18(7):e0288841. doi: 10.1371/journal.pone.0288841 (PMC10355411; doi:10.1371/journal.pone.0288841)
Supplement: S1 Fig — (PDF) [file pone.0288841.s001.pdf]

S1 Fig. Scandal Timeline

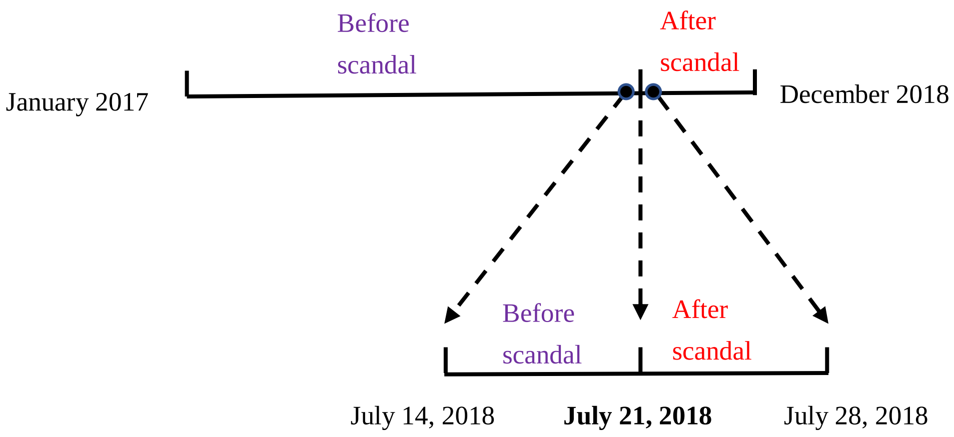

Note: The top timeline shows the entire data period from January 2017 to December 2018, with the scandal taking place in July 2018. The bottom timeline shows the subsample period for the analysis on the Baidu search index based on the daily data from July 14, 2018 to July 28, 2018. July 20, 2018 is the date when China's Jilin Provincial Medical Products Administration officially accused Changchun Changsheng Biotechnology of selling substandard DPT vaccines. As the media began to report extensively on this accusation on July 21, 2018, we consider that the event of the vaccine scandal took place on July 21, 2018.
