# Supplementary material for: Differential impacts of vaccine scandal by ethnic and socioeconomic factors: Evidence from China
Source: PLoS One. 2023 Jul 19;18(7):e0288841. doi: 10.1371/journal.pone.0288841 (PMC10355411; doi:10.1371/journal.pone.0288841)
Supplement: S2 Fig — (PDF) [file pone.0288841.s002.pdf]

S2 Figure. Population and GDP per capita Comparison between Sichuan and Other Provinces in China, 2018

Panel A. Population (Unit: million persons)

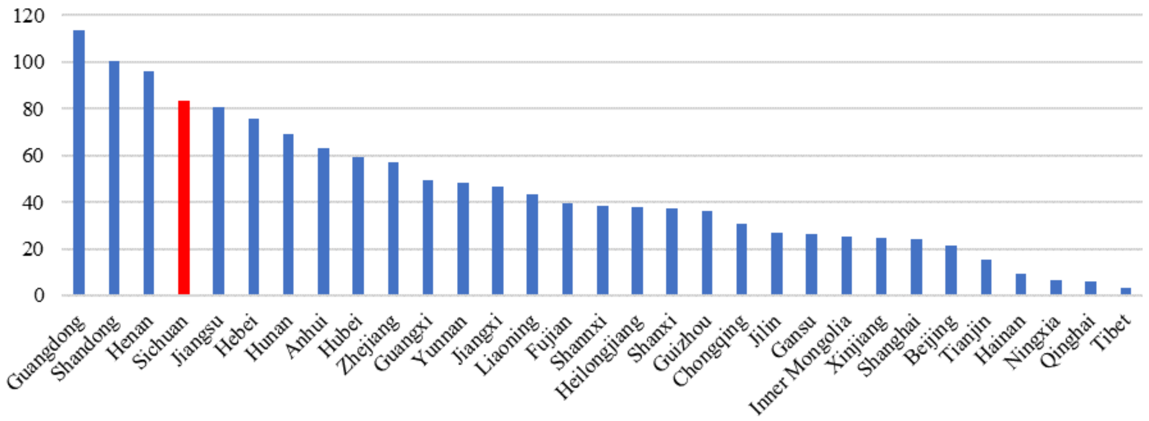

Panel B. GDP per capita (Unit: RMB per person)

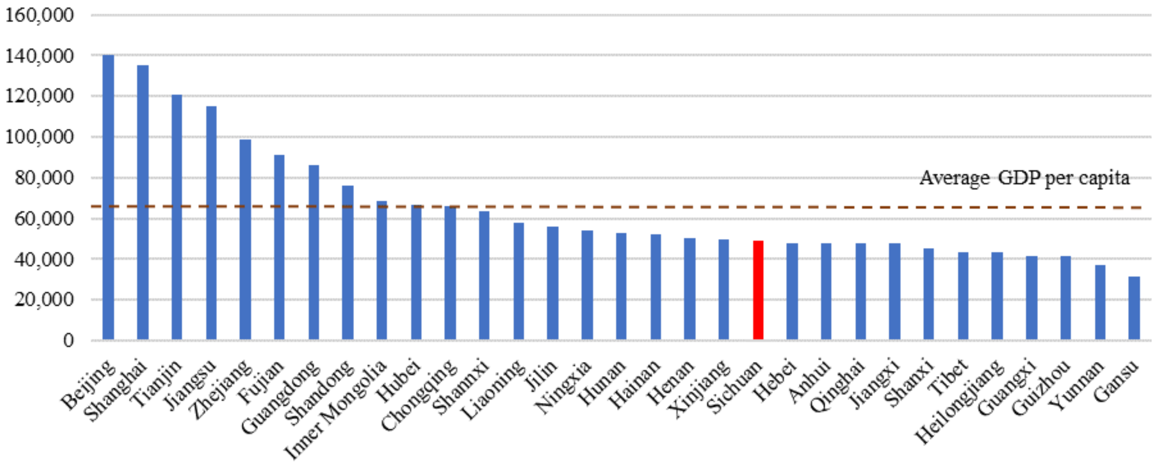

Source: China Statistical Yearbook 2019.
