# Supplementary material for: Differential impacts of vaccine scandal by ethnic and socioeconomic factors: Evidence from China
Source: PLoS One. 2023 Jul 19;18(7):e0288841. doi: 10.1371/journal.pone.0288841 (PMC10355411; doi:10.1371/journal.pone.0288841)
Supplement: S3 Fig — (PDF) [file pone.0288841.s003.pdf]

S3 Figure. Parallel Trends Assumption Check for Number of Vaccinations

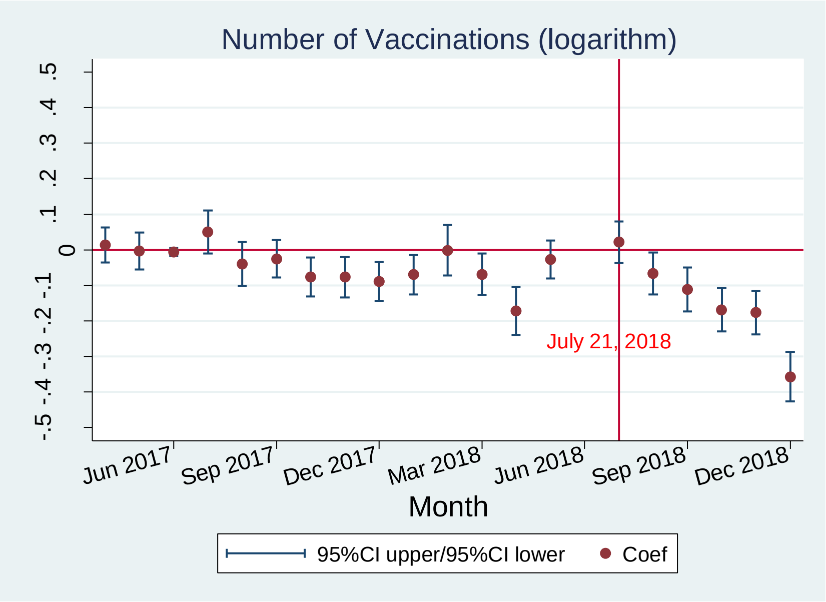

Note: This figure reports the coefficients of the model: $y_{ikt} = \sum_2^{15} \alpha_{-m} D_{it}^{-m} + \sum_0^5 \alpha_n D_{it}^{n} + \mathbf{X}_{kg}\eta + \omega_{ikt} + \theta_i + \delta_k + \mu_t + \epsilon_{ikt}$. D_{it}^{-m} and D_{it}^{n} indicate the month relative to the scandal that happened in late July 2018 for DPT vaccines. Specifically, when $-m$ ranges from -15 to -2, D_{it}^{-m} equals 1 for DPT vaccines in the pre-scandal period, and 0 otherwise; when n ranges from 0 to 5, D_{it}^{n} equals 1 for DPT vaccines in the post-scandal period, and 0 otherwise. With D_{it}^{-1} omitted, α_{-m} and α_n capture the differences in outcomes between DPT vaccines and hepatitis B vaccines relative to the difference in June 2018. Other variables are defined in the same way as in the baseline equation (1). The results indicate the comparability between DPT vaccines and hepatitis B vaccines in the pre-scandal period.
