# Supplementary material for: Differential impacts of vaccine scandal by ethnic and socioeconomic factors: Evidence from China
Source: PLoS One. 2023 Jul 19;18(7):e0288841. doi: 10.1371/journal.pone.0288841 (PMC10355411; doi:10.1371/journal.pone.0288841)
Supplement: S1 Table — (PDF) [file pone.0288841.s004.pdf]

S1 Table. The Vaccine Scandal in Numbers, 2018

Indicator	Count or Percentage
Affected provinces or cities directly under the central government	4
Involved vaccine producers	2
Doses of substandard vaccines	900,320
Changchun Changsheng Biotechnology	*499,800*[*]
Wuhan Institute of Biological Products	*400,520*[**]
Involved vaccine producers' total revenue (RMB)	
Changchun Changsheng Biotechnology	*1.55 billion*[^] *in 2017*
Wuhan Institute of Biological Products	*0.71 billion*[^^] *in 2016*
Impact on stock price of Changchun Changsheng Biotechnology	*-95%*[¶]
Number of accused government officials	*> 40*[¶¶]

Notes: Sources are as follows.

*Chinese Government Website. Available from: http://www.gov.cn/xinwen/2018-08/15/content_5314078.htm (accessed June 1, 2022).

** The State Council Information Office of the People's Republic of China. Jul 31, 2018. Available from: http://www.scio.gov.cn/xwfbh/gbwxwfbh/xwfbh/38179/Document/1635068/1635068.htm (accessed June 1, 2022).

^Sina Finance. Available from: http://vip.stock.finance.sina.com.cn/corp/view/vCB_AllBulletinDetail.php?stockid=002680&id=4110669 (accessed June 1, 2022). 1 RMB was equivalent to approximately 0.1422 U.S. dollar in 2017.

^^ Sina Finance. Jul 25, 2018. Available from: http://finance.sina.com.cn/chanjing/gsnews/2018-07-25/doc-ihftenia0815197.shtml (accessed June 1, 2022). 1 RMB was equivalent to approximately 0.1506 U.S. dollar in 2016.

¶ Shenzhen Stock Exchange. Available from: http://www.szse.cn/.

¶¶ People's Daily Online. Available from: http://politics.people.com.cn/n1/2018/0817/c1024-30233656.html.
