# Supplementary material for: Differential impacts of vaccine scandal by ethnic and socioeconomic factors: Evidence from China
Source: PLoS One. 2023 Jul 19;18(7):e0288841. doi: 10.1371/journal.pone.0288841 (PMC10355411; doi:10.1371/journal.pone.0288841)
Supplement: S2 Table — (PDF) [file pone.0288841.s005.pdf]

S2 Table. Baseline Estimation and Estimation without Vaccine Supply as Control

Number of Vaccinations (logarithm)	(1)<br>Baseline	(2)<br>Alternative
Treatment∗Post	-0.140	-0.136
	(-0.178 to -0.103)	(-0.173 to -0.098)
Vaccine supply	YES	NO
P value	0.000	0.000
Observations	7,460	8,776
R-squared	0.940	0.928

Notes: The regressions include county, vaccine type, and year-month fixed effects, as well as control for population, number of newborns, GDP per capita, and number of hospital beds per 1,000 persons. Heteroskedasticity robust standard errors are clustered at the county level. 95% confidence intervals of $Treatment_i * Post_t$ are reported in parentheses.
