# Supplementary material for: Differential impacts of vaccine scandal by ethnic and socioeconomic factors: Evidence from China
Source: PLoS One. 2023 Jul 19;18(7):e0288841. doi: 10.1371/journal.pone.0288841 (PMC10355411; doi:10.1371/journal.pone.0288841)
Supplement: S3 Table — (PDF) [file pone.0288841.s006.pdf]

S3 Table. List of Impoverished Counties in Sichuan

City/Prefecture	County/District
Aba Tibetan-Qiang autonomous prefecture	Aba, Jinchuan, Jiuzhaigou, Heishui, Hongyuan, Rangtang, Ruoergai, Songpan, Xiaojin
Bazhong	Nanjiang, Pingchang, Tongjiang,
Dazhou	Wanyuan, Xuanhan
Ganzi Tibetan autonomous prefecture	Baiyu, Batang, Danba, Daocheng, Daofu, Dege, Derong, Ganzi, Jiulong, Kangding, Litang, Luhuo, Seda, Shiqu, Xiangcheng, Xinlong, Yajiang
Guangyuan	Cangxi, Chaotian, Jiange, Qingchuan, Wangcang, Zhaohua
Leshan	Mabian
Liangshan Yi autonomous prefecture	Butong, Ganluo, Jinyang, Leibo, Meigu, Muli, Puge, Xide, Yanyuan, Yuexi, Zhaojue
Luzhou	Gulin, Xuyong
Mianyang	Pingwu
Nanchong	Langzhong
Yibin	Pingshan

Source: State Council Leading Group of Poverty Alleviation and Development (2012); Xinhua News Agency (2017); Sichuan Daily (2017).
