# Supplementary material for: Differential impacts of vaccine scandal by ethnic and socioeconomic factors: Evidence from China
Source: PLoS One. 2023 Jul 19;18(7):e0288841. doi: 10.1371/journal.pone.0288841 (PMC10355411; doi:10.1371/journal.pone.0288841)
Supplement: S4 Table — (PDF) [file pone.0288841.s007.pdf]

S4 Table. Estimation for Impoverished and Non-impoverished Counties

Number of Vaccinations (logarithm)	(1)<br>Impoverished counties	(2)<br>Non-impoverished counties
Treatment∗Post	-0.254	-0.089
	(-0.355 to -0.153)	(-0.120 to -0.059)
P value	0.000	0.000
Observations	2,185	5,275
R-squared	0.883	0.958
Z-score	-3.13 (P value 0.002)	

Notes: The regressions include county, vaccine type, and year-month fixed effects, as well as control for population, number of newborns, GDP per capita, number of hospital beds per 1,000 persons, and the vaccine supply. Heteroskedasticity robust standard errors are clustered at the county level. 95% confidence intervals of $Treatment_i * Post_t$ are reported in parentheses.
